# Longitudinal analysis of CYFRA 21-1 levels in patients with pulmonary nodules: Differential trajectories between benign and malignant cases

**DOI:** 10.1371/journal.pone.0341522

**Published:** 2026-06-23

**Authors:** Yency J. Forero, Michael N. Kammer, Kevin C. McGann, Hudson Holmes, Sheau-Chiann Chen, Heidi Chen, Samson Argaw, Timothy A. Khalil, Sanja L. Antic, Yong Zou, Lianrui Zuo, Thomas A. Lasko, Bennet A. Landman, Stephen A. Deppen, Eric L. Grogan, Fabien Maldonado

**Affiliations:** 1 Division of Allergy, Pulmonary and Critical Care Medicine, Department of Medicine, Vanderbilt University Medical Center, Nashville, Tennessee, United States of America; 2 Université Toulouse Capitole & Centre de Recherches en Cancérologie du Toulouse, Toulouse, France; 3 Department of Thoracic Surgery, Vanderbilt University Medical Center, Nashville, Tennessee, United States of America; 4 Department of Biostatistics, Department of Medicine, Vanderbilt University Medical Center, Nashville, Tennessee, United States of America; 5 Biomedical Engineering, Vanderbilt University, Nashville, Tennessee, United States of America; 6 Computer Science, Vanderbilt University, Nashville, Tennessee, United States of America; 7 Biomedical Informatics, Vanderbilt University Medical Center, Nashville, Tennessee, United States of America; 8 VA Tennessee Valley Healthcare System, Nashville, Tennessee, United States of America; Showa University Fujigaoka Hospital, JAPAN

## Abstract

**Background:**

CYFRA 21−1, a cytokeratin-19 fragment, is a validated serum biomarker for non-small cell lung cancer (NSCLC). However, most studies rely on single time-point measurements, limiting its specificity in differentiating malignancy from benign pulmonary conditions. Inspired by the clinical utility of serial PSA measurements in prostate cancer, we investigated whether longitudinal trends in CYFRA 21−1 could enhance diagnostic and monitoring capabilities in patients with pulmonary nodules.

**Methods and Findings:**

We analyzed 132 patients with pulmonary nodules from the Vanderbilt Thoracic Biorepository. For the primary analysis, patients who underwent treatment prior to biomarker assessment were excluded, resulting in an untreated cohort of 121 patients (91 benign and 30 malignant nodules). CYFRA 21−1 levels were measured serially using electrochemiluminescence assays. Longitudinal trends were assessed using linear mixed-effects models to estimate biomarker trajectories. Primary analyses compared benign vs. malignant nodules using longitudinal modeling of log-transformed CYFRA 21−1 values. At baseline, CYFRA 21−1 levels were significantly higher in malignant versus benign nodules. Longitudinal mixed-effects modeling did not demonstrate statistically significant differences in trajectories between benign and malignant nodules. Benign nodules showed a small positive trend in log(CYFRA 21−1) whereas malignant nodules showed greater longitudinal variability. The magnitude of change assessed using the absolute slope of log(CYFRA 21−1) was significantly greater in malignant nodules compared with benign nodules (p < 0.05). Exploratory diagnostic analysis showed that baseline log(CYFRA 21−1) achieved and AUC of 0.68 (95% CI 0.56-0.79) with sensitivity 0.63 and specificity 0.71. The absolute slope of log(CYFRA 21−1) yielded an AUC of 0.67 (95% CI 0.48-0.87) with sensitivity 0.39 and specificity 0.97.

**Conclusions:**

CYFRA 21−1 exhibits substantial within-patient variability over time, with trajectories that reflect disease state and treatment. These findings suggest that longitudinal monitoring of CYFRA 21−1 may provide additional information beyond single time-point measurements in the evaluation of pulmonary nodules. Further studies in large prospective cohorts are warranted to validate these findings before clinical implementation.

## Introduction

Cytokeratin-19 fragment (CYFRA 21−1) is a well-established serum biomarker that has been widely studied in lung cancer, particularly non-small cell lung cancer (NSCLC), for both diagnostic and prognostic purposes [1,2]. Many studies have evaluated CYFRA 21−1 for diagnosis or prognosis of lung cancer, either alone or in combination with other biomarkers [3–7]. Most prior studies, however, have focused on single time-point measurements (e.g., baseline levels) of CYFRA 21−1, rather than evaluating how biomarker levels evolve longitudinally over time in patients undergoing clinical evaluation for pulmonary nodules, particularly in comparison between benign and malignant nodules prior to definitive treatment. This limitation may contribute to reduced diagnostic specificity, as CYFRA 21−1 levels can also be elevated in inflammatory or other epithelial conditions, such as Chronic Obstructive Pulmonary Disease (COPD) and Interstitial Lung Fibrosis (IPF) [8]. In contrast, the clinical management of prostate cancer has long recognized the importance of biomarker trajectories. Prostate-specific antigen (PSA) “velocity” – the rate of PSA change – is an established concept used to improve early cancer detection. This concept illustrates how temporal biomarker patterns may provide clinically meaningful information beyond single measurements. For example, a rise in PSA greater than ~0.75 ng/mL per year is considered suspicious for prostate cancer, even if absolute PSA values are not yet above a static threshold [9]. Serial PSA measurements (velocity and doubling time) are routinely used to trigger biopsies, guide treatment decisions, and monitor for recurrence. This analogy suggests that tracking the temporal trajectory of a cancer biomarker can provide critical information beyond a single snapshot and helps us understand that similar longitudinal approaches could potentially improve interpretation of serum biomarkers in other malignancies.

By extension, the trajectory of CYFRA 21−1 might enhance the evaluation of pulmonary nodules. Pulmonary nodules are a common clinical dilemma, requiring differentiation between benign lesions and early lung cancer. In clinical practice, indeterminate pulmonary nodules (IPNs) are often managed with longitudinal imaging surveillance, where stability over time supports a benign diagnosis, whereas interval growth raises concern for malignancy. While a single elevated CYFRA 21−1 level can support a lung cancer diagnosis, longitudinal changes in CYFRA 21−1 could potentially signal malignancy (or benign behavior) earlier or more reliably. Recent research in lung cancer screening has begun exploring serial biomarker algorithms: repeated measurements of panels including CYFRA 21−1 have shown improved sensitivity and earlier detection of lung cancer compared to a one-time threshold approach [10]. Despite this interest, the temporal behavior of CYFRA 21−1 within individual patients for example, whether CYFRA trends upward in growing cancers or remains stable in benign nodules remains understudied.

Here we present a longitudinal analysis of CYFRA 21−1 in patients with pulmonary nodules, comparing biomarker trajectories between benign and malignant nodules using repeated measurements and mixed effects modeling. We aimed to determine whether malignant nodules demonstrate distinct longitudinal CYFRA 21−1 trajectories compared with benign lesions.

## Methods

### Study Cohort

Patients with pulmonary nodules were identified from the Vanderbilt University Thoracic Biorepository. This prospective collection with retrospective blinded evaluation (PROBE) study analyzed serum samples from patients with pulmonary nodules who underwent serial blood sampling during their clinical evaluation. Because this study represents an exploratory analysis using available biorepository samples, a formal prospective power calculation was not performed. Instead, the sample size reflects all eligible patients with available longitudinal CYFRA 21−1 measurements within the cohort during the study period. Serum was processed and stored within 2 hours of blood draw, according to the Early Detection Research Network’s Lung Clinical Validation Center standard operating protocol [11]. All patients were eventually categorized as having either benign or malignant nodules based on definitive diagnoses. Malignant nodules were confirmed by histopathology. Benign nodules were defined either by histopathologic confirmation of non-malignant tissue or by radiographic stability on serial CT imaging for at least 24 months, consistent with established pulmonary surveillance guidelines form the Fleischner Society and the American College of Chest Physicians [12].

These imaging data allowed classification of nodules as benign or malignant and supported the longitudinal evaluation of nodule behavior throughout the follow up period.

History of previous cancer refers to a prior diagnosis of any malignancy before enrollment in the study cohort, regardless of cancer type of treatment status.

### CYFRA 21−1 measurement

Samples were obtained from the VUMC Thoracic Biorepository^11^ and accessed for research purposes between 01/07/2008–31/12/2018. Serum CYFRA 21–1 concentrations were measured using the Roche Elecsys Cobas e411 analyzer (electrochemiluminescence immunoassay), following the manufacturer’s protocol, with a College of American Pathologist (CAPP) certified laboratory. The CYFRA 21–1 assay demonstrated high analytical precision, with reported coefficients of variation typically ranging between approximately 1–2% for repeatability and below 5% for intermediate precision. The lower limit of detection for the assay is reported as <0.10 ng/mL. All samples were run concurrently, in a randomized order and in a blind fashion.

### Statistical analysis

Demographic, clinical characteristics and biomarker were summarized overall and by group of interest. Continuous variables will be described using means and standard deviations or medians and interquartile ranges. Categorical variables will be summarized using frequencies and percentages. Linear mixed-effects models (LME) were used to analyze longitudinal trends in CYFRA 21−1 while accounting for repeated measures within individual patients. Time, diagnostic group, and their interaction were included as fixed effect with patient ID included as a random intercept to account for baseline differences between individuals. This allowed estimation of group-specific trends while accounting for repeated measures within individuals.

CYFRA 21−1 values were log-transformed prior to modeling, due to the right-skewed distribution of raw concentrations. The primary analysis was restricted to benign versus untreated malignant nodules to address the study’s diagnostic objective and to avoid confounding from treatment related biomarker changes. Measurementes obtained after treatment or surgical resection (“treated cancer”) were excluded from the primary models and analyzed separately as exploratory analyses to evaluate biomarker changes following intervention. removal.

Time was defined as the duration (in days) from each patient’s first CYFRA 21−1 measurement (baseline) to Each subsequent measurement. The LME model was fitted using maximum likelihood estimation. Type III ANOVA F-tests were used to assess the significance of fixed effects. Post-hoc pairwise comparisons of estimated marginal means were conducted using Wald test with emmeans package, with Tukey adjustment, and results were back transformed to the original scale with 95% confidence intervals.

To quantify the magnitude of longitudinal biomarker, change, individual slopes of log transformed CYFRA 21−1 values over time were estimated for each patient using simple linear regression models. The absolute value of these slopes was then calculated to represent the magnitude of change in CYFRA 21−1 levels over time, regardless of direction. These absolute slopes were subsequently compared across diagnostics groups using one-way ANOVA as an exploratory analysis.

To quantify the rate of biomarker changes over time, we estimated the slope of biomarker trajectory for each patient using simple linear regression. Specifically, the biomarker CYFRA 21−1 concentration was analyzed on the natural logarithmic scale to stabilize variance and approximate linear change over time. For each patient i, the following model was fitted:


$$\begin{array}{c} Log\;(CYFRA_{it})\;=\;\beta_{\text{0}}\;+\beta_{\text{1}}\;x\;timeit\;+\;\varepsilon_{it} \end{array}$$

where CYFRA𝑖𝑡 is the biomarker value at observation time 𝑡, and 𝛽_1_ represents the temporal rate of change in log(CYFRA). 𝜀𝑖𝑡 is an error term assumed to follow a normal distribution with mean 0 and variance 𝜎2. The estimated regression coefficient ${\hat{𝛽}}_{1}$ was taken as the slope describing the biomarker trajectory. To capture the magnitude of change regardless of direction (increase or decrease), the absolute value of the slope (i.e., ${\hat{𝛽}}_{1}$) was used in the analysis.

To improve clinical interpretability, we therefore conducted an exploratory diagnostic analysis. Specifically, we summarized each subject’s longitudinal biomarker profile using (1) the baseline log(CYFRA) and (2) the individual absolute slope of log(CYFRA) and (3) the individual absolute slope of log(CYFRA). Logistic regression was used to evaluate association between biomarker and disease group (benign vs untreated cancer). Discriminatory performance for distinguishing benign from untreated cancer was evaluated using receiver operating characteristic (ROC) analysis. The area under the ROC curve (AUC) was reported with 95% confidence intervals. Optimism-adjusted AUC with 95% bootstrap confidence interval using the.632 correction was also reported. Sensitivity and specificity were calculated using an exploratory cutoff defined by the Younden index.

All statistical analyses were performed in R (v4.5.1), with α = 0.05 as the significance threshold (two-tailed).

### Ethical considerations

The study was conducted according to the Declaration of Helsinki. The study protocol was approved by the Institutional Review Board (IRB) of Vanderbilt University Medical Center (protocol number IRB#030763). Written informed consent was obtained from all participants, and all methods were carried out in accordance with applicable institutional and regulatory guidelines.

## Results

### Cohort description

A total of 132pulmonary nodules were included in the Vanderbilt Thoracic Biorepository cohort. Among these, 91 patients had benign nodules and 41 had malignant nodules based on definitive diagnosis. For the primary analysis, patients who underwent treatment or surgery were excluded to avoid confounding due to treatment related biomarker changes. The resulting untreated cohort included 121 patients (91 benign and 30 malignant) (Table 1).

**Table 1:** Summary of Patients Characteristics.

			Group		
Characteristic	N	Overall N = 1211	Benign N = 911	Malignant N = 301	p-value2
**Age**	121				0.505
Mean (SD)		63.5 (5.1)	63.2 (4.7)	64.3 (6.2)	
Median (Q1, Q3)		62.6 (59.5, 67.1)	62.6 (59.5, 66.6)	62.9 (59.4, 68.2)	
**Sex**	121				0.796
Male		75 (62.0%)	57 (62.6%)	18 (60.0%)	
Female		46 (38.0%)	34 (37.4%)	12 (40.0%)	
**Race**	121				0.758
Caucasian		115 (95.0%)	85 (93.4%)	30 (100.0%)	
African American		4 (3.3%)	4 (4.4%)	0 (0.0%)	
Asian		1 (0.8%)	1 (1.1%)	0 (0.0%)	
Native American		1 (0.8%)	1 (1.1%)	0 (0.0%)	
**Smokers**	121				0.248
Ever-smoker		120 (99.2%)	91 (100.0%)	29 (96.7%)	
Never-smoker		1 (0.8%)	0 (0.0%)	3 (3.3%)	
**Pack Years**	120				0.813
Mean (SD)		55.5 (25.8)	53.7 (23.7)	61.1 (31.4)	
Median (Q1, Q3)		47.5 (40.0, 64.8)	47.0 (40.2, 62.0)	50.0 (42.0, 83.2)	
**History of previous cancer**	121	41 (33.9%)	28 (30.8%)	13 (43.3%)	0.0207
**Lung Nodule Location** ^**3**^	112				
RLL		21 (18.8%)	13 (15.9%)	8 (26.7%)	
RUL		27 (24.4%)	20 (24.4%)	7 (23.3%)	
LLL		19 (17.0%)	11 (13.4%)	8 (26.7%)	
LUL		12(10.7%)	6 (7.3%)	6 (20.0%)	
RML		6 (5.4%)	6 (7.3%)	0 (0.0%)	
Right Hilum		1 (0.9%)	0 (0.0%)	1 (3.3%)	
Left Hilum		0 (0.0%)	0 (0.0%)	1 (2.4%)	
Insignificant nodule or data entry error		26 (23.2%)	26 (31.7%)	0 (0.0%)	
**Histology diagnosis**	119				<0.001
Adenocarcinoma		14 (11.8%)	0 (0.0%)	14 (46.7%)	
Large Cell Neuroendocrine		2 (1.7%)	0 (0.0%)	2 (6.7%)	
Negative for Malignant Cells		12 (10.1%)	12 (13.5%)	0 (0.0%)	
No Diagnosis		2 (1.7%)	2 (2.2%)	0 (0.0%)	
Non-Small Cell (NSCLC)		2 (1.7%)	0 (0.0%)	2 (6.7%)	
Non-lung primary		1 (0.8%)	0 (0.0%)	1 (3.3%)	
Normal		73 (61.3%)	73 (82.0%)	0 (0.0%)	
Other – cancer		2 (1.7%)	0 (0.0%)	2 (6.7%)	
Squamous Cell Carcinoma		9(7.6%)	0 (0.0%)	9 (30.0%)	
Squamous Metaplasia		1 (0.8%)	1 (1.1%)	0 (0.0%)	
Unsatisfactory		1 (0.8%)	1 (1.1%)	0 (0.0%)	
**Lung cancer history**	121	6 (5.0%)	2 (2.2%)	4 (13.3%)	0.033

^1^n (%)

^2^Wilcoxon rank sum test; Pearson’s Chi-squared test; Fisher’s exact test

^3^Right Upper Lobe (RUL), Right Lower Lobe (RLL), Left Upper Lobe (LUL), Left Lower Lobe (LLL).

The two groups were similar in age, sex, race, smoking status, pack -years, and lung nodule location. However, history of previous cancer and prior lung cancer were more frequent among patients with malignant nodules. As expected, histological diagnosis differed significantly between groups.

An exploratory subgroup analysis included 16 malignant patients with biomarker measurements available both before and after surgical or therapeutical intervention (Table 2)

**Table 2:** Clinical, pathological and outcome characteristics of patients with confirmed malignancy and who underwent surgical resection.

Clinical Stage	Pathological Stage	Histology	Location^1^	Metastasis	Cancer History	Survival Status	Time to Event (months) (death, alive or last follow up
IIIA	T2N2M0	Squamous cell carcinoma	LLL	No	No	Unknown	12
IIIA	T2N2M0	Adenocarcinoma	LLL	Yes (CNS)	No	Deceased	15
IB	T1N0M0	Squamous cell carcinoma	LUL	Yes (bone)	No	Deceased	77
IIA	T1aN1M0	Adenocarcinoma	RLL	Yes (CNS)	No	Alive	104
IIA	T1aN1M0	Adenocarcinoma	LUL	Yes (hilar)	No	Deceased	24
IB	T2aN0M0	Squamous cell carcinoma	RLL	Yes (hilar)	No	Deceased	91
IA	T1bN0M0	Neuroendocrine	LLL	No	No	Alive	77
IIIA	T3N1M0	Adenocarcinoma	RLL	Unknown	No	Deceased	39
IB	T1bN0M0	Adenocarcinoma	LUL	Unknown	No	Unknown	80
IIIA		Squamous cell carcinoma	Lymph node(4R)	Yes (bone)	Yes (colon)	Deceased	38
IIB	T3N0M0	Adenocarcinoma	RLL	No	No	Unknown	37
IIA	T1bN0M0	Neuroendocrine	RUL	No	No	Alive	101
N/A	N/A	Metastatic adenocarcinoma	LUL	Yes	Yes (Ampullar adenocarcinoma)	Deceased	26
IIIB	T4N3M0	Adenocarcinoma	LLL	Yes	No	Unknown	29
IIB	T3N0M0	Squamous cell carcinoma	RUL	No	No	Unknown	4

^1^Right Upper Lobe (RUL), Right Lower Lobe (RLL), Left Upper Lobe (LUL), Left Lower Lobe (LLL).

None of the benign cases underwent surgical removal of the nodule during the study; these patients were followed with serial observations alone. By contrast, the malignant cases in the surgical subgroup had detailed staging information and contributed to the serial CYFFRA 21−1 measurements both before and after surgical resection of the tumor, providing internal “pre vs post” comparisons. Five of the cancer patients had only a single CYFRA 21−1 measurement (at the initial visit, with no follow-up sample, these were patients who, for example, were lost to follow-up or had immediate treatment elsewhere). The remaining cancer patients had multiple serial measurements in at least one phase (pre- or post-treatment). The median follow-up duration for biomarker measurements was approximately 6–12 months, with a median of 3 blood draws per patient (range 1–9, as some patients in the paired group had frequent follow-up draws). Notably, CYFRA 21−1 levels were generally low in benign patients across all time points, though one benign-case patient exhibited an outlier high value (peak CYFRA 11.3 ng/mL) despite ultimately having a benign diagnosis. This was an exceptional case; most benign nodules had CYFRA levels well below the typical diagnostic cut-off of 3.3 ng/mL.

### Baseline characteristics and biomarker level

The two groups were similar with respect to age, sex, race, smoking status, pack years and lung nodule location (p < 0.05). Most patients were Caucasian, and the majority had a history of smoking. Nodule distribution across lung lobes was comparable between groups. However, the history of previous cancer and prior lung cancer was more frequent among patients with malignant nodules (p < 0.05) for both comparisons. Histological diagnosis differed significantly between groups, as expected (p < 0.001).

The baseline comparisons were restricted to untreated malignant cases to align with the primary diagnostic objective of the study.

### Diagnostic performance of baseline CYFRA 21−1

The diagnostic performance of baseline CYFRA 21−1 for differentiation benign from malignant nodules was evaluated using logistic regression.

**Fig 1:**
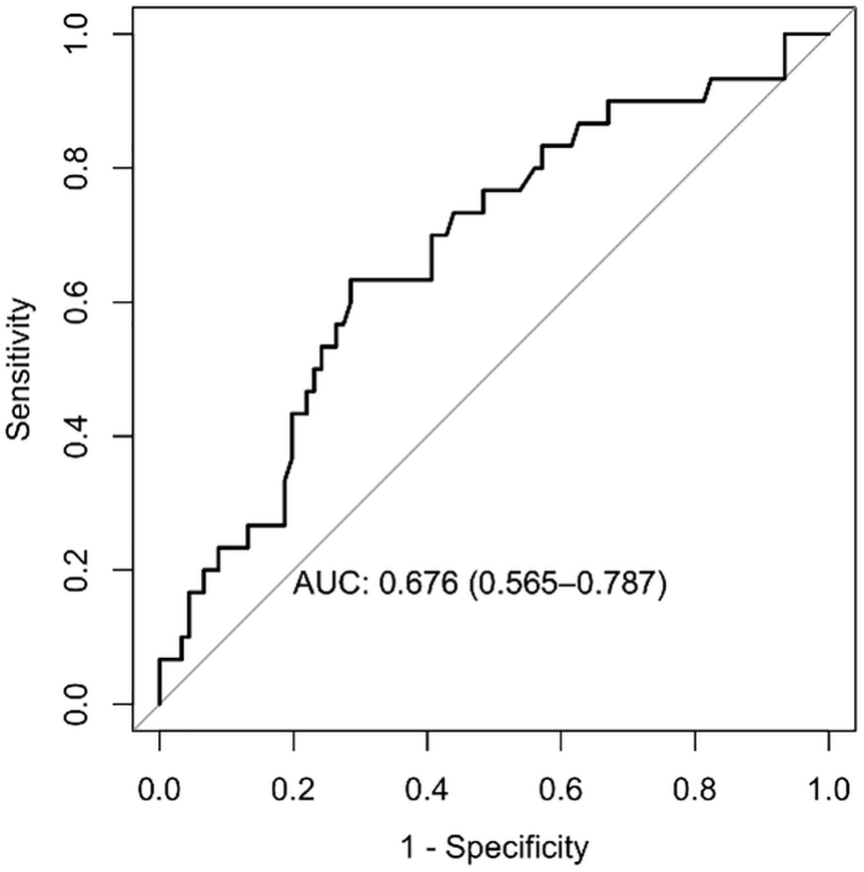
Receiver Operating Characteristic (ROC) curve for baseline CYFRA 21−1 in the discrimination of benign versus untreated malignant pulmonary nodules. The area under the curve (AUC) was 0.676 (95% CI: 0.565–0.787). The diagonal grey line represents the reference line of no discrimination. The optimal Youden index threshold yielded a sensitivity of 0.633 and a specificity of 0.714.

Baseline CYFRA 21−1 demonstrated modest discriminative ability, with an area under the curve (AUC) of 0.68 (95% CI 0.565-0.787). Using the optimal Youden index threshold, sensitivity was 0.633 and specificity was 0.714 (Table 3, Fig 1).

**Table 3:** Diagnostic performance of baseline CYFRA 21−1 and longitudinal metrics for distinguishing benign vs untreated malignant nodules.

	AUC (95% CI)^c^	Optimistic adjusted AUC (95% CI)^d^	Youden cut off	Sensitivity	Specificity
**Baseline log(CYFRA)** ^**a**^	0.68 (0.56, 0.79)	0.67 (0.58, 0.76)	0.811	0.633	0.714
**Absolute slope of log(CYFRA)** ^**b**^	0.67 (0.48, 0.87)	–	0.03	0.385	0.967

a. 91 Benign subjects, 30 cancer subjects.

b. 91 Benign Subjects, 13 cancer subjects, each with more than two nodules.

c. AUC with 95% confidence

d. Optimism-adjusted AUC with 95% bootstrap confidence interval using the.632 correction. Due to small sample size, optimism-adjusted AUC for absolute slope of log(CYFRA) was not applicable

***These findings should be interpreted in the context of the relatively small sample size

### Longitudinal changes in CYFRA 21−1 levels (mixed effect model)

**Fig 2:**
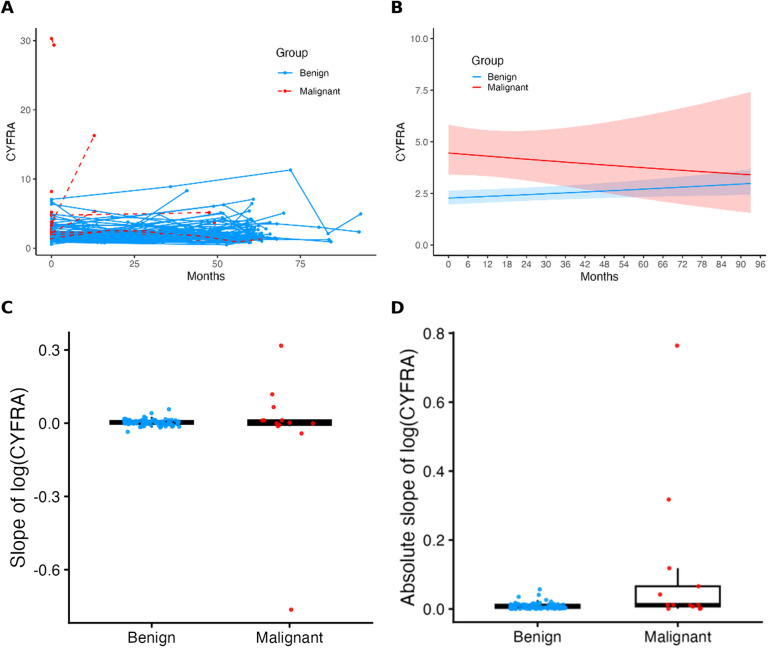
Longitudinal CYFRA 21−1 dynamics in patients with benign (blue) and untreated malignant (red) pulmonary nodules. **(A)** Individual spaghetti plot showing serial CYFRA 21−1 measurements over time (months) for each participant. Solid blue lines represent benign cases; dashed red lines represent malignant cases. **(B)** Linear mixed-effects model–estimated group trajectories with 95% confidence bands, demonstrating higher overall CYFRA 21−1 levels in malignant nodules (mean 2.67 ng/mL) compared with benign nodules (mean 1.87 ng/mL), without a significant time-by-group interaction (p = 0.21). **(C)** Distribution of individual patient slopes of log(CYFRA 21−1) per month, by diagnostic group. No significant difference was observed between groups (p = 0.35; AUC 0.47). **(D)** Distribution of the absolute value of individual slopes of log(CYFRA 21−1) per month, by diagnostic group. Absolute slopes were numerically higher in malignant nodules, though the difference did not reach statistical significance (p = 0.16; AUC 0.60, 95% CI 0.48–0.87).

Longitudinal CYFRA 21–1 measurements were observed from 91 subjects with benign nodules and 30 with untreated cancer (Fig 2A). Changes in CYFRA 21–1 over time were evaluated using a linear mixed effect model (LME) including time and malignancy status as fixed effects and patient level random intercepts. Across the untreated cohort (121 subjects, contributing 397 measurements), malignant nodules were associated with significantly higher overall CYFRA 21–1 levels compared with benign nodules (p < 0.001). On average, patients with untreated malignancy nodules had higher CYFRA 21–1 levels (2.67 ng/mL CI 2.16-3.29) than those with benign nodules (1.87 ng/mL, 95% CI 1.71-2.05). However, the interaction between time and malignancy status was not statistically significant (p = 0.21) suggesting that longitudinal trends over time were similar between groups (Fig 2B).

### Individual slope analysis

To evaluate subject specific biomarker dynamics, slopes of CYFRA 21−1 (Fig 2C) were estimated for patients with sufficient longitudinal measurements prior to treatment. This analysis included 104 subjects (91 benign and 13 malignant). The estimated slope of CYFRA 21−1 over time did not significantly differentiate benign from malignant nodules (p = 0.35). Discriminatory performance was limited to AUC 0.47.

### Absolute slope analysis

To better capture variability in biomarker dynamics, the absolute slope of CYFRA 21−1 (Fig. 2D) was analyzed. However, absolute slope was higher in malignant nodules compared to benign nodules: however, this difference did not reach statistical significance (p = 0.16), with an AUC of 0.60 (95% CI 0.48−.87).

## Discussion

In this study, we evaluated the longitudinal behavior of the serum biomarker CYFRA 21−1 in patients with pulmonary nodules, focusing on comparison between benign and untreated malignant nodules as the primary diagnostic analysis, while separately assessing post-treatment changes as an exploratory analysis. To our knowledge, this represents one of the few studies specifically examining within-patient CYFRA trajectories over time in this clinical context.

Our findings demonstrate that patients with malignant nodules exhibit higher CYFRA 21−1 levels compared with those with benign nodules, both at baseline and across longitudinal measurements, This observation is consistent with prior literature supporting the diagnostic role of CYFRA 21−1 in lung cancer [1–5]. However, despite these differences in absolute levels, we did not observe statistically significant differences in longitudinal trajectories between benign and malignant nodules, as reflected by the non-significant time by group interaction and slope-based analyses. These findings suggest that, within the limitations of this cohort, CYFRA 21−1 differs between groups primarily in magnitude rather than in the rate of change over time. The lack of significant differences in slope-based analyses may be explained by several factors. First the number of malignant cases with sufficient longitudinal measurements prior to treatment was limited, reducing statistical power. Second, many malignant cases underwent early intervention, thereby truncating the natural history of untreated tumor related biomarker dynamics. Additionally, heterogeneity in tumor biology and treatment approaches may have contributed to variability in longitudinal patterns.

**Fig 3:**
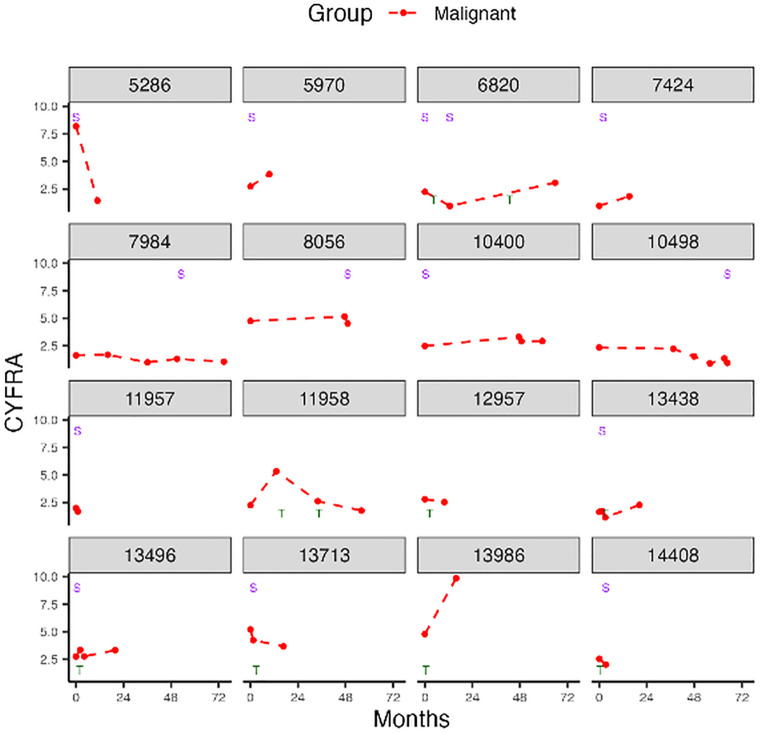
Individual CYFRA 21−1 trajectories for each malignant patient in the exploratory pre- and post-treatment subgroup (n = 16). Each panel displays serial CYFRA 21−1 measurements (ng/mL, y-axis) over time in months (x-axis) for a single patient identified by study ID. Dashed red lines connect longitudinal measurements. Annotations indicate the timing of surgery (S, purple) or other therapeutic intervention (T, green) relative to sample collection. Substantial interindividual variability was observed; overall, a trend toward lower CYFRA 21−1 levels was noted following intervention, consistent with reduction in tumor burden.

The exploratory analysis of patients with pre- and post-treatment measurements (n = 16) demonstrated a general trend toward lower CYFRA 21−1 levels following intervention. Median CYFRA 21−1 levels decreased from 2.64 ng/mL preoperatively to 1.94 ng/mL postoperatively. However, substantial interindividual variability was observed, with some patients showing minimal change or slight increases following treatment. These findings are consistent with prior studies demonstrating that CYFRA 21−1 reflects tumor burden and may decrease following treatment [6,7], although they should be interpreted cautiously given the small sample size (Fig 3).

The concept of longitudinal biomarker assessment has been well established in other malignancies, such as prostate cancer, where PSA velocity and doubling time provide clinically meaningful information beyond single measurements [9]. While a similar framework may be appliable to CYFRA 21−1, our findings suggest that, in this dataset, longitudinal changes alone may not provide sufficient discriminatory value to differentiate benign from malignant nodules.

Importantly, overlap in CYFRA 21−1 levels between benign and malignant nodules was observed, highlighting a known limitation of single biomarker measurements. Elevated CYFRA 21−1 levels in benign cases may reflect underlying inflammatory or epithelial conditions, such as chronic obstructive pulmonary disease (COPD) or interstitial lung disease [8], although these factors were not systematically assessed in this cohort.

Recent studies have suggested that incorporating biomarker trajectories may improve early detection of lung cancer [10]. However, in our study, trajectory – based metrics such as slope and absolute slope showed limited discriminatory performance, indicating that longitudinal patterns alone may not be sufficient without integration of additional clinical or imaging data.

Several limitations of our study should be acknowledged. The sample size, especially for the malignant cases with longitudinal data, was limited, affecting statistical power and the stability of slope-based estimates. The follow up duration was relatively short, and measurement frequencies varied between patients. Additionally, some benign nodules were classified based on imaging follow-up rather than histopathology, introducing potential misclassification. Finally, potential confounders such as comorbidities, tumor subtype, and renal function were not accounted for.

Despite these limitations, this study provides clinically relevant insights into the longitudinal behavior of CYFRA 21−1. While absolute CYFRA 21−1 levels remain useful for distinguishing malignant from benign nodules, longitudinal trajectories alone may have limited added diagnostic value in this setting. Future prospective studies with larger cohorts and integrated biomarker models are needed to further define the role of serial CYFRA 21−1 measurements in clinical practice.

## Conclusions

In summary, this longitudinal pilot study highlights that while CYFRA 21−1 has long been recognized as a lung cancer biomarker, its behavior over time provides additional insights into tis clinical utility CYFRA 21−1 levels were consistently higher in malignant nodules compared with benign nodules, supporting its role as a diagnostic biomarker. However, longitudinal changes over time, including slope-based analyses, did not significantly differentiate benign from malignant nodules in this cohort. Exploratory analyses suggested a decrease in CYFRA 21−1 levels following treatment in a subset of patients, although these findings were variable and limited by small sample size. Overall, these findings indicate that while absolute CYFRA 21−1 levels may aid in distinguishing malignant from benign nodules, longitudinal patterns alone may have limited discriminatory value in this setting.

Larger prospective studies are needed to further evaluate the potential role of serial CYFRA 21−1 measurements in lung nodule assessment and clinical decision making.
