# Supplementary material for: Longitudinal analysis of CYFRA 21-1 levels in patients with pulmonary nodules: Differential trajectories between benign and malignant cases
Source: PLoS One. 2026 Jun 23;21(6):e0341522. doi: 10.1371/journal.pone.0341522 (PMC13289921; doi:10.1371/journal.pone.0341522)
Supplement: Attachment — Submitted filename: Response to Reviewers Plos One.docx [file pone.0341522.s002.docx]

**RESPONSE TO REVIEWERS: MANUSCRIPT ID: PONE-D-26-01020**

We sincerely thank the editor and reviewers for their careful evaluation of our manuscript and for their insightful and constructive comments. We have carefully addressed all suggestions and believe that the revision has significantly improved the clarity, rigor and overall quality of the manuscript.

All changes in the manuscript have been clearly indicated and are referenced with corresponding page in line number.

**Reviewer 1**

**1. If the primary aim is to differentiate benign from malignant nodules, the treated cancer group reflects a different clinical question (treatment-response monitoring) and may confound interpretation. Please present the main analysis using benign vs untreated cancer only.**

We agree that inclusion of treated cancer cases may confound the primary diagnostic objective of distinguishing benign from malignant nodules. In response, we have revised the manuscript to clearly restrict the primary analysis to benign versus untreated malignant nodules, while analyses involving treated cancer cases are now explicitly presented as exploratory:

Specifically:

- The Methods section has been updated to define the primary comparison as benign versus untreated cancer.
- The results section now clearly describes the untreated cohort used for the main analyses.
- All primary statistical models (baseline comparison, mixed-effects models and diagnostic performance analyses) were conducted withing the untreated cohort.
- Analyses involving post treatment measurements are now presented separately as exploratory.

**2. Please specify the follow-up duration and criteria used to classify nodules as benign based on imaging surveillance. Because benign classification based solely on follow-up cannot fully exclude malignancy, please explicitly discuss the risk of misclassification as a study limitation.**

We explained the criteria used to define benign nodules in:

- Methods section: stating that benign nodules were defined either by histopathologic confirmation or by radiographic stability on serial CT imaging for at least 24 months, consistent with established pulmonary nodule surveillance guidelines.
- Discussion: we explicitly acknowledge the potential for misclassification when benign status is determined based on imaging follow up rather than histopathology.

**3. The current conclusions rely on qualitative impressions of “mild vs steep” changes, which limits clinical interpretability. Please report diagnostic performance for benign vs untreated cancer, such as ROC-AUC and sensitivity/specificity using an exploratory cutoff.**

We agree that reporting quantitative diagnostic performance metrics improves clinical interpretability. In response, we have expanded the:

- Results: we include ROC-AUC, sensitivity and specificity for the primary diagnostic comparison between benign and untreated malignant. Specifically, baseline CYFRA 21-1 demonstrated an AUC of 0.676 (95% CI 0.565-0.787), with sensitivity of 0.633 and specificity of 0.714 using an exploratory cutoff determined by the Younden index.
- Methods: we clarify in this section that ROC analyses were performed, and that sensitivity and specificity were calculated using a data driven cutoff (Younden index)

To improve clinical interpretability, we therefore conducted an exploratory diagnostic analysis. Specifically, we summarized each subject’s longitudinal biomarker profile using (1) the baseline Log(CYFRA) and (2) the individual slope of log(CYFRA). We then evaluated their ability to discriminate benign from untreated cancer usin ROC analysis. The area under the curve (AUC) with 95% confidence intervals

|  | **AUC (95% CI)^c^** | **Optimistic adjusted AUC (95% CI)^d^** | **Younden cut off** | **Sensitivity** | **Specificity** |
| --- | --- | --- | --- | --- | --- |
| **Baseline log(CYFRA)^a^** | 0.68 (0.56, 0.79) | 0.67 (0.58, 0.76) | 0.811 | 0.633 | 0.714 |
| **Absolute slope of log(CYFRA)^b^** | 0.67 (0.48, 0.87) | - | 0.03 | 0.385 | 0.96.7 |

a: 91 benign subjects, 30 cancer subjects.

b: 91 benign subjects, 13 cancer subjects, each with more than two nodules.

c: AUC with 95% confidence

d: Optimism-adjusted AUC with 95% bootstrap confidence interval using the .632 correction. Due to small sample size, optimism-adjusted AUC for absolute slope of log(CYFRA) was not applicable

**4. The figures are not visible or are too unclear to interpret. Please ensure all figures are submitted with adequate format and resolution.**

All figures have been regenerated with improved resolution and clarity. Figure legends have been revised to enhance interpretability.

**Reviewer 2**

**1. The rationale for the sample size is unclear. The author should clarify the statistical power for 132 patients in this study.**

This study represents an exploratory analysis based on a biorepository cohort. Therefore, no formal a prior sample size or power calculation was performed. Instead, we included all eligible patients with available longitudinal CYFRA 21-1 measurements during the study period, resulting in a total sample size of 132 patients.

We clarified this point

- Methods: we clarified and explicitly stated that the analysis in exploratory and hypothesis generating rather than powered for definitive inference.

**2. The definition of “absolute slope of log-transformed CYFRA values” is unclear (Page13, Line262). A clearer explanation is needed as to why this value was used for statistical analysis.**

To quantify the rate of biomarker, change over time, we estimated the slope of the biomarker trajectory for each patient using simple linear regression. Specifically, the biomarker CYFRA concentration was analyzed on the natural logarithmic scale to stabilize variance and approximate linear change over time. For each patient i, the following model was fitted:

Log (CYFRA*_it_*) = β_0_ + β_1_ x time*_it_* + ε*_it’_*

where CYFRA𝑖𝑡 is the biomarker value at observation time 𝑡, and 𝛽1represents the temporal rate of change in log(CYFRA). 𝜀𝑖𝑡 is an error term assumed to follow a normal distribution with mean 0 and variance 𝜎2. The estimated regression coefficient 𝛽̂1was taken as the slope describing the biomarker trajectory.To capture the magnitude of change regardless of direction (increase or decrease), the absolute value of the slope (i.e., |𝛽̂1|) was used in the analysis.

We clarify this point in the Methods section.

**3. The authors described “If a patient’s CYFRA remains high or rebounds after surgery, it could signal residual disease or early recurrence” (Page16, Line330). With only 16 surgical cases, this statement is an exaggeration.**

In response, we have revised both the Results and Discussion sections to clearly describe these findings as exploratory and descriptive, rather than definitive.

Specifically:

- Results: The post treatment analysis was labeled as exploratory, language suggesting causal or definitive effects has been removed and emphasize the observed variability across patients.
- Discussion: this section has been revised to clarify that these results cannot establish a definitive relationship between tumor resection and biomarker dynamics.

These changes align the interpretation with the limitations of the data and address the reviewer’s concern.

**4. In the Limitations section, the author should describe the potential for selection bias in the surgical cases. Furthermore, it should be noted that the lack of standardization in clinical staging poses a clinical issue.**

We have expanded the Discussion section to explain these issues, specifically:

- We know that the surgical subgroup may be subject to selection bias, as patients undergoing resection likely represent a subset with operable disease and may not be representative of the broader population of patients with pulmonary nodules.
- We also acknowledge that clinical staging was not standardized across all cases due to the retrospective nature of the study, which may introduce additional heterogeneity in the interpretation of results.

**5. In the Limitations section, the impact of the storage period of biorepository samples on measurement results should be described.**

- Discussion we have added this item as study limitation. Specifically, we now state that although samples were processed using standardized biorepository protocols, variability in storage duration may have influenced measured CYFRA 21- levels.
- Methods: we also retained the description of standardized sample handling procedures to provide context regarding pre-analytical quality control.

**6. In the Conclusions section, “CYFRA 2101 velocity or doubling time could enhance lung nodule risk stratification” (Page19, Line 415), I think this sentence is too much because the sample size of the data in this study was small. The author should make more cautious assertions.**

We have revised the section to avoid overinterpretation of the findings, particularly regarding post treatment biomarker changes. Specifically:

- Conclusion: we now emphasize that the clinical utility of longitudinal CYFRA 21-1 trajectories remains uncertain. We state also that further validation in larger prospective cohorts is required before clinical implementation.

**7. The resolution of Figure 1 is too low to be clearly discernible. It should be replaced with a figure that readers can understand.**

All figures have been regenerated with improved resolution and clarity. Figure legends have been revised to enhance interpretability.

**Reviewer 3**

**Introduction**

**1. It appears that previous studies addressing longitudinal follow-up of biomarkers—both in comparisons between cancer and benign cases and in pre- versus post-surgical settings—have not been sufficiently reviewed in the manuscript.**

We have revised the introduction to include a brief discussion of prior studies that have evaluated longitudinal changes in CYFRA 21-1, particularly in the context of treatment response and disease progression. We also clarify that most prior work has focused on treated populations, highlighting the gap addressed by our study, which focuses on untreated pulmonary nodules.

**Methods**

**Study Cohort**

**2. Line 126-129 & line 207-208: The authors state that benign cases were confirmed through “long-term imaging follow-up,” yet the description lacks sufficient detail. The manuscript should clearly specify the enrollment criteria, including the exact duration of follow-up required to define benignity. In addition, the study should present detailed information on how these patients’ data were structured and documented throughout the follow-up period.**

We have clarified the criteria specifically, benign nodules were defined either by histopathologic confirmation or by radiographic stability on serial CT imaging for a minimum follow up period of 24 months, consistent with established guidelines.

**3. Line 130-135: Given that the study’s conclusions rely on longitudinal changes and slope differences in CYFRA 21-1 levels, detailed analytical validation of the assay is essential. Information regarding functional sensitivity (CV20), limit of detection, intra- and inter-assay coefficients of variation, and quality control procedures should be provided to ensure that observed biomarker fluctuations exceed analytical variability. Results - Most results are suggestive but preliminary, and the evidence remains insufficient to establish longitudinal CYFRA 21-1 as a reliable diagnostic tool without external validation**

In response, we have revised the Discussion and Conclusion sections to adopt a more cautious tone, emphasizing the exploratory nature of the analysis, the limited sample size and the need for validation in larger prospective cohorts before clinical applications. We also included information regarding functional sensitivity (CV20) and quality control procedures for the biomarker CYFRA 21-1.

**4. Line 177 & Table 1: ‘history of previous cancer’ was not fully explained and more detailed should be added in the manuscript**

We added a definition in the Methods section specifying that this variable refers to a prior diagnosis of any malignancy before enrollment in the study cohort, regardless of cancer type of treatment status.

**5. Line 190-192, 207-208: The statement, “Benign nodules included 73 normal tissue samples,” may inadvertently imply that benign status was confirmed surgically, which could mislead readers into assuming postoperative histopathologic confirmation. This wording should be revised for clarity. Based on the study description, it appears that most benign nodules were diagnosed through imaging follow-up (e.g., serial CT surveillance) rather than surgical resection or biopsy confirmation, and this distinction should be explicitly stated in the manuscript.**

We have revised the Results section to clarify that benign nodules were classified based on either histopathologic confirmation or radiographic stability on longitudinal imaging follow up, with the majority of cases defined using imaging criteria rather than surgical confirmation. In addition, we ensure consistency in reporting cohort numbers following exclusion of treated cases in the primary analysis.

**6. Line 222~224: Specific data supporting this statement were not provided. It is unclear whether the data presented in lines 226–229 correspond to this point, and clarification from the authors would be necessary.**

We agree that clearer linkage between statements in the Results section and the supporting data is important. In response, we have revised the Results section to explicitly reference the corresponding table (Table 1 and Table 2) to ensure that all statements are directly supported by the presented data. In addition, we included specific numerical results (p values, AUC, sensitivity, specificity) and direct references to the relevant figures Table 3 and Figure 1).

**7. Line 235~238: Contrary to the authors’ strong assertions in the manuscript, the differences observed between pre- and post-surgical measurements do not appear to be substantial. Moreover, the Discussion section does not provide sufficient explanation or interpretation of this finding.**

We have avoided overinterpretation of these findings. The pre-post analysis is now clearly presented as exploratory, and the language has been softened to emphasize variability across patients and the limited sample size. We also clarified that these results are descriptive and do not establish a definitive relationship between tumor resection and biomarker dynamics.

We also revised the Discussion section to better contextualize these findings and explicitly acknowledge their limitations.

**Discussion**

**8. Line 348~354: Details regarding “concomitant inflammatory lung conditions” should be provided on a patient-by-patient basis (or at least in a clearly defined subgroup analysis), including the specific diagnoses and clinical context. As written, the manuscript repeatedly attributes CYFRA increases to presumed inflammation, but offers no supporting clinical data or detailed explanation for why CYFRA truly rose in these cases, relying largely on speculation throughout.**

The Discussion and Conclusion sections we avoided speculative interpretation and to better reflect the exploratory nature of these findings. We no longer attribute CYFRA elevations to specific inflammatory conditions and instead present these observations more cautiously, emphasizing that potential explanations remain speculative due to the lack of detailed clinical data.

**9. Line 395-400: The results of this study do not sufficiently support this statement and appear to be based largely on speculation rather than robust evidence.**

We have softened the language throughout and avoided definitive or causal statements, emphasizing the limited sample size, lack of statistical significance in some analyses and the need for external validations.
