# Supplementary material for: Longitudinal analysis of CYFRA 21-1 levels in patients with pulmonary nodules: Differential trajectories between benign and malignant cases
Source: PLoS One. 2026 Jun 23;21(6):e0341522. doi: 10.1371/journal.pone.0341522 (PMC13289921; doi:10.1371/journal.pone.0341522)
Supplement: Peer review history

# Peer review: Decision Letter 0

**DOI:** 10.1371/journal.pone.0341522.r001

**Authors:** Fumihiro Yamaguchi

23 Feb 2026

PONE-D-26-01020

Longitudinal Analysis of CYFRA 21-1 Levels in Patients with Pulmonary Nodules: Differential Trajectories Between Benign and Malignant Cases and Impact of Tumor Resection

PLOS One

Dear Dr. Forero,

Thank you for submitting your manuscript to PLOS ONE. After careful consideration, we feel that it has merit but does not fully meet PLOS ONE’s publication criteria as it currently stands. Therefore, we invite you to submit a revised version of the manuscript that addresses the points raised during the review process.

Please submit your revised manuscript by Apr 09 2026 11:59PM. If you will need more time than this to complete your revisions, please reply to this message or contact the journal office at plosone@plos.org. When you're ready to submit your revision, log on to https://www.editorialmanager.com/pone/ and select the 'Submissions Needing Revision' folder to locate your manuscript file.

Please include the following items when submitting your revised manuscript:

- A letter that responds to each point raised by the academic editor and reviewer(s). You should upload this letter as a separate file labeled 'Response to Reviewers'.
- A marked-up copy of your manuscript that highlights changes made to the original version. You should upload this as a separate file labeled 'Revised Manuscript with Track Changes'.
- An unmarked version of your revised paper without tracked changes. You should upload this as a separate file labeled 'Manuscript'.

If you would like to make changes to your financial disclosure, please include your updated statement in your cover letter. Guidelines for resubmitting your figure files are available below the reviewer comments at the end of this letter.

If applicable, we recommend that you deposit your laboratory protocols in protocols.io to enhance the reproducibility of your results. Protocols.io assigns your protocol its own identifier (DOI) so that it can be cited independently in the future. For instructions see: https://journals.plos.org/plosone/s/submission-guidelines#loc-laboratory-protocols. Additionally, PLOS ONE offers an option for publishing peer-reviewed Lab Protocol articles, which describe protocols hosted on protocols.io. Read more information on sharing protocols at https://plos.org/protocols?utm_medium=editorial-email&utm_source=authorletters&utm_campaign=protocols.

We look forward to receiving your revised manuscript.

Kind regards,

Fumihiro Yamaguchi

Academic Editor

PLOS One

**Journal Requirements:**

When submitting your revision, we need you to address these additional requirements.

1. Please ensure that your manuscript meets PLOS ONE's style requirements, including those for file naming. The PLOS ONE style templates can be found at

https://journals.plos.org/plosone/s/file?id=wjVg/PLOSOne_formatting_sample_main_body.pdf and

https://journals.plos.org/plosone/s/file?id=ba62/PLOSOne_formatting_sample_title_authors_affiliations.pdf

2. Please note that PLOS One has specific guidelines on code sharing for submissions in which author-generated code underpins the findings in the manuscript. In these cases, we expect all author-generated code to be made available without restrictions upon publication of the work. Please review our guidelines at https://journals.plos.org/plosone/s/materials-and-software-sharing#loc-sharing-code and ensure that your code is shared in a way that follows best practice and facilitates reproducibility and reuse.

3. Thank you for stating the following financial disclosure:

“NIH National Cancer Institute Grants: 1R01CA253923 to Fabien Maldonado

U01CA152662 to Eric L. Grogan”

Please state what role the funders took in the study.  If the funders had no role, please state: "The funders had no role in study design, data collection and analysis, decision to publish, or preparation of the manuscript."

If this statement is not correct you must amend it as needed.

Please include this amended Role of Funder statement in your cover letter; we will change the online submission form on your behalf.

4. Please note that funding information should not appear in any section or other areas of your manuscript. We will only publish funding information present in the Funding Statement section of the online submission form. Please remove any funding-related text from the manuscript.

5. In the online submission form, you indicated that your data is available only on request from a third party. Please note that your Data Availability Statement is currently contact details for the third party, such as an email address or a link to where data requests can be made. Please update your statement with the missing information.

6. In the online submission form you indicate that your data is not available for proprietary reasons and have provided a contact point for accessing this data. Please note that your current contact point is a co-author on this manuscript. According to our Data Policy, the contact point must not be an author on the manuscript and must be an institutional contact, ideally not an individual. Please revise your data statement to a non-author institutional point of contact, such as a data access or ethics committee, and send this to us via return email. Please also include contact information for the third party organization, and please include the full citation of where the data can be found.

7. Please upload a new copy of Figures 1 and 2 as the detail is not clear. Please follow the link for more information:  https://journals.plos.org/plosone/s/figures

8. If the reviewer comments include a recommendation to cite specific previously published works, please review and evaluate these publications to determine whether they are relevant and should be cited. There is no requirement to cite these works unless the editor has indicated otherwise.

**Additional Editor Comments:**

The reviewers have recommended publication, but also suggest significant revisions to your manuscript.  Therefore, I invite you to respond to the reviewers' comments and revise your manuscript.

[Note: HTML markup is below. Please do not edit.]

Reviewers' comments:

Reviewer's Responses to Questions

**Comments to the Author**

1. Is the manuscript technically sound, and do the data support the conclusions?

The manuscript must describe a technically sound piece of scientific research with data that supports the conclusions. Experiments must have been conducted rigorously, with appropriate controls, replication, and sample sizes. The conclusions must be drawn appropriately based on the data presented.

Reviewer #1: Yes

Reviewer #2: Partly

Reviewer #3: No

**********

2. Has the statistical analysis been performed appropriately and rigorously?

Reviewer #1: I Don't Know

Reviewer #2: Yes

Reviewer #3: No

**********

3. Have the authors made all data underlying the findings in their manuscript fully available?

The PLOS Data policy requires authors to make all data underlying the findings described in their manuscript fully available without restriction, with rare exception (please refer to the Data Availability Statement in the manuscript PDF file). The data should be provided as part of the manuscript or its supporting information, or deposited to a public repository. For example, in addition to summary statistics, the data points behind means, medians and variance measures should be available. If there are restrictions on publicly sharing data—e.g. participant privacy or use of data from a third party—those must be specified.

Reviewer #1: Yes

Reviewer #2: Yes

Reviewer #3: No

**********

4. Is the manuscript presented in an intelligible fashion and written in standard English?

PLOS ONE does not copyedit accepted manuscripts, so the language in submitted articles must be clear, correct, and unambiguous. Any typographical or grammatical errors should be corrected at revision, so please note any specific errors here.

Reviewer #1: Yes

Reviewer #2: Yes

Reviewer #3: Yes

**********

5. Review Comments to the Author

Please use the space provided to explain your answers to the questions above. You may also include additional comments for the author, including concerns about dual publication, research ethics, or publication ethics. (Please upload your review as an attachment if it exceeds 20,000 characters)

Reviewer #1: This manuscript addresses an important and clinically relevant question by shifting the focus from a single CYFRA 21-1 value to its longitudinal trajectory in patients evaluated for pulmonary nodules. The attempt to quantify temporal patterns using longitudinal modeling is meaningful and has potential to inform how tumor markers might be interpreted for nodule assessment and follow-up.

Major comments

1. If the primary aim is to differentiate benign from malignant nodules, the treated cancer group reflects a different clinical question (treatment-response monitoring) and may confound interpretation. Please present the main analysis using benign vs untreated cancer only.

2. Please specify the follow-up duration and criteria used to classify nodules as benign based on imaging surveillance. Because benign classification based solely on follow-up cannot fully exclude malignancy, please explicitly discuss the risk of misclassification as a study limitation.

3. The current conclusions rely on qualitative impressions of “mild vs steep” changes, which limits clinical interpretability. Please report diagnostic performance for benign vs untreated cancer, such as ROC-AUC and sensitivity/specificity using an exploratory cutoff.

4. The figures are not visible or are too unclear to interpret. Please ensure all figures are submitted with adequate format and resolution.

Reviewer #2: Dear authors,

Thank you for submitting your manuscript to PLOS One. I appreciate the opportunity to review it. The paper, “Longitudinal Analysis of CYFRA 21-1 Levels in Patients with Pulmonary Nodules: Differential Trajectories Between Benign and Malignant Cases and Impact of Tumor Resection”, which was a single-center, prospective collection with retrospective blinded evaluation study analyzed serum samples from a cohort of patients with pulmonary nodules. I think the manuscript would be worthy for publication of the journal after the major revision mentioned below.

1. The rationale for the sample size is unclear. The author should clarify the statistical power for 132 patients in this study.

2. The definition of “absolute slope of log-transformed CYFRA values” is unclear (Page13, Line262). A clearer explanation is needed as to why this value was used for statistical analysis.

3. The authors described “If a patient’s CYFRA remains high or rebounds after surgery, it could signal residual disease or early recurrence” (Page16, Line330). With only 16 surgical cases, this statement is an exaggeration.

4. In the Limitations section, the author should describe the potential for selection bias in the surgical cases. Furthermore, it should be noted that the lack of standardization in clinical staging poses a clinical issue.

5. In the Limitations section, the impact of the storage period of biorepository samples on measurement results should be described.

6. In the Conclusions section, “CYFRA 2101 velocity or doubling time could enhance lung nodule risk stratification” (Page19, Line 415), I think this sentence is too much because the sample size of the data in this study was small. The author should make more cautious assertions.

7. The resolution of Figure 1 is too low to be clearly discernible. It should be replaced with a figure that readers can understand.

I hope my review would help the authors to improve the manuscript. Thank you again for your submission.

Best wishes,

Reviewer #3: This paper investigates the longitudinal dynamics of serum CYFRA 21-1 levels in patients with pulmonary nodules, comparing biomarker trajectories between benign and malignant cases. Using repeated measurements and linear mixed-effects modeling, the study demonstrates that malignant nodules exhibit distinct temporal patterns and greater variability than benign lesions, with trends suggesting biomarker decline following tumor resection. The authors propose that serial CYFRA 21-1 monitoring may provide additional diagnostic value beyond single time-point measurements, although further validation in larger cohorts is required.

Contrary to the original intention, the actual study results do not sufficiently support the investigators’ key proposal or hypothesis, and the findings lack detailed explanation and in-depth interpretation. In particular, the low quality of the figures makes it difficult to accurately interpret and assess the underlying data.

Introduction

- It appears that previous studies addressing longitudinal follow-up of biomarkers—both in comparisons between cancer and benign cases and in pre- versus post-surgical settings—have not been sufficiently reviewed in the manuscript.

Methods

Study Cohort

- Line 126-129 & line 207-208: The authors state that benign cases were confirmed through “long-term imaging follow-up,” yet the description lacks sufficient detail. The manuscript should clearly specify the enrollment criteria, including the exact duration of follow-up required to define benignity. In addition, the study should present detailed information on how these patients’ data were structured and documented throughout the follow-up period.

- Line 130-135: Given that the study’s conclusions rely on longitudinal changes and slope differences in CYFRA 21-1 levels, detailed analytical validation of the assay is essential. Information regarding functional sensitivity (CV20), limit of detection, intra- and inter-assay coefficients of variation, and quality control procedures should be provided to ensure that observed biomarker fluctuations exceed analytical variability.

Results

- Most results are suggestive but preliminary, and the evidence remains insufficient to establish longitudinal CYFRA 21-1 as a reliable diagnostic tool without external validation

- Line 177 & Table 1: ‘history of previous cancer’ was not fully explained and more detailed should be added in the manuscript

- Line 190-192, 207-208: The statement, “Benign nodules included 73 normal tissue samples,” may inadvertently imply that benign status was confirmed surgically, which could mislead readers into assuming postoperative histopathologic confirmation. This wording should be revised for clarity. Based on the study description, it appears that most benign nodules were diagnosed through imaging follow-up (e.g., serial CT surveillance) rather than surgical resection or biopsy confirmation, and this distinction should be explicitly stated in the manuscript.

- Line 222~224: Specific data supporting this statement were not provided. It is unclear whether the data presented in lines 226–229 correspond to this point, and clarification from the authors would be necessary.

- Line 235~238: Contrary to the authors’ strong assertions in the manuscript, the differences observed between pre- and post-surgical measurements do not appear to be substantial. Moreover, the Discussion section does not provide sufficient explanation or interpretation of this finding.

Discussion

- Line 348~354: Details regarding “concomitant inflammatory lung conditions” should be provided on a patient-by-patient basis (or at least in a clearly defined subgroup analysis), including the specific diagnoses and clinical context. As written, the manuscript repeatedly attributes CYFRA increases to presumed inflammation, but offers no supporting clinical data or detailed explanation for why CYFRA truly rose in these cases, relying largely on speculation throughout.

- Line 395-400: The results of this study do not sufficiently support this statement and appear to be based largely on speculation rather than robust evidence.

**********

6. PLOS authors have the option to publish the peer review history of their article (what does this mean?). If published, this will include your full peer review and any attached files.

If you choose “no”, your identity will remain anonymous but your review may still be made public.

**Do you want your identity to be public for this peer review?**   For information about this choice, including consent withdrawal, please see our Privacy Policy.

Reviewer #1: No

Reviewer #2: No

Reviewer #3: No

**********

[NOTE: If reviewer comments were submitted as an attachment file, they will be attached to this email and accessible via the submission site. Please log into your account, locate the manuscript record, and check for the action link "View Attachments". If this link does not appear, there are no attachment files.]

To ensure your figures meet our technical requirements, please review our figure guidelines: https://journals.plos.org/plosone/s/figures

You may also use PLOS’s free figure tool, NAAS, to help you prepare publication quality figures: https://journals.plos.org/plosone/s/figures#loc-tools-for-figure-preparation.

NAAS will assess whether your figures meet our technical requirements by comparing each figure against our figure specifications.

# Author comment: Author response to Decision Letter 1

**DOI:** 10.1371/journal.pone.0341522.r002

13 Apr 2026

RESPONSE TO REVIEWERS: MANUSCRIPT ID: PONE-D-26-01020

We sincerely thank the editor and reviewers for their careful evaluation of our manuscript and for their insightful and constructive comments. We have carefully addressed all suggestions and believe that the revision has significantly improved the clarity, rigor and overall quality of the manuscript.

All changes in the manuscript have been clearly indicated and are referenced with corresponding page in line number.

Reviewer 1

1. If the primary aim is to differentiate benign from malignant nodules, the treated cancer group reflects a different clinical question (treatment-response monitoring) and may confound interpretation. Please present the main analysis using benign vs untreated cancer only.

We agree that inclusion of treated cancer cases may confound the primary diagnostic objective of distinguishing benign from malignant nodules. In response, we have revised the manuscript to clearly restrict the primary analysis to benign versus untreated malignant nodules, while analyses involving treated cancer cases are now explicitly presented as exploratory:

Specifically:

• The Methods section has been updated to define the primary comparison as benign versus untreated cancer.

• The results section now clearly describes the untreated cohort used for the main analyses.

• All primary statistical models (baseline comparison, mixed-effects models and diagnostic performance analyses) were conducted withing the untreated cohort.

• Analyses involving post treatment measurements are now presented separately as exploratory.

2. Please specify the follow-up duration and criteria used to classify nodules as benign based on imaging surveillance. Because benign classification based solely on follow-up cannot fully exclude malignancy, please explicitly discuss the risk of misclassification as a study limitation.

We explained the criteria used to define benign nodules in:

• Methods section: stating that benign nodules were defined either by histopathologic confirmation or by radiographic stability on serial CT imaging for at least 24 months, consistent with established pulmonary nodule surveillance guidelines.

• Discussion: we explicitly acknowledge the potential for misclassification when benign status is determined based on imaging follow up rather than histopathology.

3. The current conclusions rely on qualitative impressions of “mild vs steep” changes, which limits clinical interpretability. Please report diagnostic performance for benign vs untreated cancer, such as ROC-AUC and sensitivity/specificity using an exploratory cutoff.

We agree that reporting quantitative diagnostic performance metrics improves clinical interpretability. In response, we have expanded the:

• Results: we include ROC-AUC, sensitivity and specificity for the primary diagnostic comparison between benign and untreated malignant. Specifically, baseline CYFRA 21-1 demonstrated an AUC of 0.676 (95% CI 0.565-0.787), with sensitivity of 0.633 and specificity of 0.714 using an exploratory cutoff determined by the Younden index.

• Methods: we clarify in this section that ROC analyses were performed, and that sensitivity and specificity were calculated using a data driven cutoff (Younden index)

To improve clinical interpretability, we therefore conducted an exploratory diagnostic analysis. Specifically, we summarized each subject’s longitudinal biomarker profile using (1) the baseline Log(CYFRA) and (2) the individual slope of log(CYFRA). We then evaluated their ability to discriminate benign from untreated cancer usin ROC analysis. The area under the curve (AUC) with 95% confidence intervals

AUC (95% CI)c Optimistic adjusted AUC (95% CI)d Younden cut off Sensitivity Specificity

Baseline log(CYFRA)a 0.68 (0.56, 0.79) 0.67 (0.58, 0.76) 0.811 0.633 0.714

Absolute slope of log(CYFRA)b 0.67 (0.48, 0.87) - 0.03 0.385 0.96.7

a: 91 benign subjects, 30 cancer subjects.

b: 91 benign subjects, 13 cancer subjects, each with more than two nodules.

c: AUC with 95% confidence

d: Optimism-adjusted AUC with 95% bootstrap confidence interval using the .632 correction. Due to small sample size, optimism-adjusted AUC for absolute slope of log(CYFRA) was not applicable

4. The figures are not visible or are too unclear to interpret. Please ensure all figures are submitted with adequate format and resolution.

All figures have been regenerated with improved resolution and clarity. Figure legends have been revised to enhance interpretability.

Reviewer 2

1. The rationale for the sample size is unclear. The author should clarify the statistical power for 132 patients in this study.

This study represents an exploratory analysis based on a biorepository cohort. Therefore, no formal a prior sample size or power calculation was performed. Instead, we included all eligible patients with available longitudinal CYFRA 21-1 measurements during the study period, resulting in a total sample size of 132 patients.

We clarified this point

• Methods: we clarified and explicitly stated that the analysis in exploratory and hypothesis generating rather than powered for definitive inference.

2. The definition of “absolute slope of log-transformed CYFRA values” is unclear (Page13, Line262). A clearer explanation is needed as to why this value was used for statistical analysis.

To quantify the rate of biomarker, change over time, we estimated the slope of the biomarker trajectory for each patient using simple linear regression. Specifically, the biomarker CYFRA concentration was analyzed on the natural logarithmic scale to stabilize variance and approximate linear change over time. For each patient i, the following model was fitted:

Log (CYFRAit) = β0 + β1 x timeit + εit’

where CYFRA𝑖𝑡 is the biomarker value at observation time 𝑡, and 𝛽1represents the temporal rate of change in log(CYFRA). 𝜀𝑖𝑡 is an error term assumed to follow a normal distribution with mean 0 and variance 𝜎2. The estimated regression coefficient ${\hat{𝛽}}_{1}$ was taken as the slope describing the biomarker trajectory.To capture the magnitude of change regardless of direction (increase or decrease), the absolute value of the slope (i.e., |${\hat{𝛽}}_{1}$|) was used in the analysis.

We clarify this point in the Methods section.

3. The authors described “If a patient’s CYFRA remains high or rebounds after surgery, it could signal residual disease or early recurrence” (Page16, Line330). With only 16 surgical cases, this statement is an exaggeration.

In response, we have revised both the Results and Discussion sections to clearly describe these findings as exploratory and descriptive, rather than definitive.

Specifically:

• Results: The post treatment analysis was labeled as exploratory, language suggesting causal or definitive effects has been removed and emphasize the observed variability across patients.

• Discussion: this section has been revised to clarify that these results cannot establish a definitive relationship between tumor resection and biomarker dynamics.

These changes align the interpretation with the limitations of the data and address the reviewer’s concern.

4. In the Limitations section, the author should describe the potential for selection bias in the surgical cases. Furthermore, it should be noted that the lack of standardization in clinical staging poses a clinical issue.

We have expanded the Discussion section to explain these issues, specifically:

• We know that the surgical subgroup may be subject to selection bias, as patients undergoing resection likely represent a subset with operable disease and may not be representative of the broader population of patients with pulmonary nodules.

• We also acknowledge that clinical staging was not standardized across all cases due to the retrospective nature of the study, which may introduce additional heterogeneity in the interpretation of results.

5. In the Limitations section, the impact of the storage period of biorepository samples on measurement results should be described.

• Discussion we have added this item as study limitation. Specifically, we now state that although samples were processed using standardized biorepository protocols, variability in storage duration may have influenced measured CYFRA 21- levels.

• Methods: we also retained the description of standardized sample handling procedures to provide context regarding pre-analytical quality control.

6. In the Conclusions section, “CYFRA 2101 velocity or doubling time could enhance lung nodule risk stratification” (Page19, Line 415), I think this sentence is too much because the sample size of the data in this study was small. The author should make more cautious assertions.

We have revised the section to avoid overinterpretation of the findings, particularly regarding post treatment biomarker changes. Specifically:

• Conclusion: we now emphasize that the clinical utility of longitudinal CYFRA 21-1 trajectories remains uncertain. We state also that further validation in larger prospective cohorts is required before clinical implementation.

7. The resolution of Figure 1 is too low to be clearly discernible. It should be replaced with a figure that readers can understand.

All figures have been regenerated with improved resolution and clarity. Figure legends have been revised to enhance interpretability.

Reviewer 3

Introduction

1. It appears that previous studies addressing longitudinal follow-up of biomarkers—both in comparisons between cancer and benign cases and in pre- versus post-surgical settings—have not been sufficiently reviewed in the manuscript.

We have revised the introduction to include a brief discussion of prior studies that have evaluated longitudinal changes in CYFRA 21-1, particularly in the context of treatment response and disease progression. We also clarify that most prior work has focused on treated populations, highlighting the gap addressed by our study, which focuses on untreated pulmonary nodules.

Methods

Study Cohort

2. Line 126-129 & line 207-208: The authors state that benign cases were confirmed through “long-term imaging follow-up,” yet the description lacks sufficient detail. The manuscript should clearly specify the enrollment criteria, including the exact duration of follow-up required to define benignity. In addition, the study should present detailed information on how these patients’ data were structured and documented throughout the follow-up period.

We have clarified the criteria specifically, benign nodules were defined either by histopathologic confirmation or by radiographic stability on serial CT imaging for a minimum follow up period of 24 months, consistent with established guidelines.

3. Line 130-135: Given that the study’s conclusions rely on longitudinal changes and slope differences in CYFRA 21-1 levels, detailed analytical validation of the assay is essential. Information regarding functional sensitivity (CV20), limit of detection, intra- and inter-assay coefficients of variation, and quality control procedures should be provided to ensure that observed biomarker fluctuations exceed analytical variability. Results - Most results are suggestive but preliminary, and the evidence remains insufficient to establish longitudinal CYFRA 21-1 as a reliable diagnostic tool without external validation

In response, we have revised the Discussion and Conclusion sections to adopt a more cautious tone, emphasizing the exploratory nature of the analysis, the limited sample size and the need for validation in larger prospective cohorts before clinical applications. We also included information regarding functional sensitivity (CV20) and quality control procedures for the biomarker CYFRA 21-1.

4. Line 177 & Table 1: ‘history of previous cancer’ was not fully explained and more detailed should be added in the manuscript

We added a definition in the Methods section specifying that this variable refers to a prior diagnosis of any malignancy before enrollment in the study cohort, regardless of cancer type of treatment status.

5. Line 190-192, 207-208: The statement, “Benign nodules included 73 normal tissue samples,” may inadvertently imply that benign status was confirmed surgically, which could mislead readers into assuming postoperative histopathologic confirmation. This wording should be revised for clarity. Based on the study description, it appears that most benign nodules were diagnosed through imaging follow-up (e.g., serial CT surveillance) rather than surgical resection or biopsy confirmation, and this distinction should be explicitly stated in the manuscript.

We have revised the Results section to clarify that benign nodules were classified based on either histopathologic confirmation or radiographic stability on longitudinal imaging follow up, with the majority of cases defined using imaging criteria rather than surgical confirmation. In addition, we ensure consistency in reporting cohort numbers following exclusion of treated cases in the primary analysis.

6. Line 222~224: Specific data supporting this statement were not provided. It is unclear whether the data presented in lines 226–229 correspond to this point, and clarification from the authors would be necessary.

We agree that clearer linkage between statements in the Results section and the supporting data is important. In response, we have revised the Results section to explicitly reference the corresponding table (Table 1 and Table 2) to ensure that all statements are directly supported by the presented data. In addition, we included specific numerical results (p values, AUC, sensitivity, specificity) and direct references to the relevant figures Table 3 and Figure 1).

7. Line 235~238: Contrary to the authors’ strong assertions in the manuscript, the differences observed between pre- and post-surgical measurements do not appear to be substantial. Moreover, the Discussion section does not provide sufficient explanation or interpretation of this finding.

We have avoided overinterpretation of these findings. The pre-post analysis is now clearly presented as exploratory, and the language has been softened to emphasize variability across patients and the limited sample size. We also clarified that these results are descriptive and do not establish a definitive relationship between tumor resection and biomarker dynamics.

We also revised the Discussion section to better contextualize these findings and explicitly acknowledge their limitations.

Discussion

8. Line 348~354: Details regarding “concomitant inflammatory lung conditions” should be provided on a patient-by-patient basis (or at least in a clearly defined subgroup analysis), including the specific diagnoses and clinical context. As written, the manuscript repeatedly attributes CYFRA increases to presumed inflammation, but offers no supporting clinical data or detailed explanation for why CYFRA truly rose in these cases, relying largely on speculation throughout.

The Discussion and Conclusion sections we avoided speculative interpretation and to better reflect the exploratory nature of these findings. We no longer attribute CYFRA elevations to specific inflammatory conditions and instead present these observations more cautiously, emphasizing that potential explanations remain speculative due to the lack of detailed clinical data.

9. Line 395-400: The results of this study do not sufficiently support this statement and appear to be based largely on speculation rather than robust evidence.

We have softened the language throughout and avoided definitive or causal statements, emphasizing the limited sample size, lack of statistical significance in some analyses and the need for external validations.

Attachment Submitted filename: Response to Reviewers Plos One.docx

# Peer review: Decision Letter 1

**DOI:** 10.1371/journal.pone.0341522.r003

**Authors:** Fumihiro Yamaguchi, Fumihiro Yamaguchi

14 May 2026

Longitudinal Analysis of CYFRA 21-1 levels in patients with pulmonary nodules: differential trajectories between benign and malignant cases.

PONE-D-26-01020R1

Dear Dr. Forero,

We’re pleased to inform you that your manuscript has been judged scientifically suitable for publication and will be formally accepted for publication once it meets all outstanding technical requirements.

Within one week, you’ll receive an e-mail detailing the required amendments. When these have been addressed, you’ll receive a formal acceptance letter and your manuscript will be scheduled for publication.

An invoice will be generated when your article is formally accepted. Please note, if your institution has a publishing partnership with PLOS and your article meets the relevant criteria, all or part of your publication costs will be covered. Please make sure your user information is up-to-date by logging into Editorial Manager at Editorial Manager® and clicking the ‘Update My Information' link at the top of the page. For questions related to billing, please contact billing support.

If your institution or institutions have a press office, please notify them about your upcoming paper to help maximize its impact. If they’ll be preparing press materials, please inform our press team as soon as possible -- no later than 48 hours after receiving the formal acceptance. Your manuscript will remain under strict press embargo until 2 pm Eastern Time on the date of publication. For more information, please contact onepress@plos.org.

Kind regards,

Fumihiro Yamaguchi

Academic Editor

PLOS One

Additional Editor Comments (optional):

Reviewers' comments:

Reviewer's Responses to Questions

**Comments to the Author**

1. If the authors have adequately addressed your comments raised in a previous round of review and you feel that this manuscript is now acceptable for publication, you may indicate that here to bypass the “Comments to the Author” section, enter your conflict of interest statement in the “Confidential to Editor” section, and submit your "Accept" recommendation.

Reviewer #1: All comments have been addressed

Reviewer #2: (No Response)

**********

2. Is the manuscript technically sound, and do the data support the conclusions?

The manuscript must describe a technically sound piece of scientific research with data that supports the conclusions. Experiments must have been conducted rigorously, with appropriate controls, replication, and sample sizes. The conclusions must be drawn appropriately based on the data presented.

Reviewer #1: Yes

Reviewer #2: (No Response)

**********

3. Has the statistical analysis been performed appropriately and rigorously?

Reviewer #1: Yes

Reviewer #2: (No Response)

**********

4. Have the authors made all data underlying the findings in their manuscript fully available?

The PLOS Data policy requires authors to make all data underlying the findings described in their manuscript fully available without restriction, with rare exception (please refer to the Data Availability Statement in the manuscript PDF file). The data should be provided as part of the manuscript or its supporting information, or deposited to a public repository. For example, in addition to summary statistics, the data points behind means, medians and variance measures should be available. If there are restrictions on publicly sharing data—e.g. participant privacy or use of data from a third party—those must be specified.

Reviewer #1: Yes

Reviewer #2: (No Response)

**********

5. Is the manuscript presented in an intelligible fashion and written in standard English?

PLOS ONE does not copyedit accepted manuscripts, so the language in submitted articles must be clear, correct, and unambiguous. Any typographical or grammatical errors should be corrected at revision, so please note any specific errors here.

Reviewer #1: Yes

Reviewer #2: (No Response)

**********

6. Review Comments to the Author

Please use the space provided to explain your answers to the questions above. You may also include additional comments for the author, including concerns about dual publication, research ethics, or publication ethics. (Please upload your review as an attachment if it exceeds 20,000 characters)

Reviewer #1: (No Response)

Reviewer #2: (No Response)

**********

7. PLOS authors have the option to publish the peer review history of their article (what does this mean?). If published, this will include your full peer review and any attached files.

If you choose “no”, your identity will remain anonymous but your review may still be made public.

**Do you want your identity to be public for this peer review?**   For information about this choice, including consent withdrawal, please see our Privacy Policy.

Reviewer #1: No

Reviewer #2: No

**********

# Editor report: Acceptance letter

**DOI:** 10.1371/journal.pone.0341522.r004

**Authors:** Fumihiro Yamaguchi, Fumihiro Yamaguchi

PONE-D-26-01020R1

PLOS One

Dear Dr. Forero,

I'm pleased to inform you that your manuscript has been deemed suitable for publication in PLOS One. Congratulations! Your manuscript is now being handed over to our production team.

At this stage, our production department will prepare your paper for publication. This includes ensuring the following:

* All references, tables, and figures are properly cited

* All relevant supporting information is included in the manuscript submission,

* There are no issues that prevent the paper from being properly typeset

You will receive further instructions from the production team, including instructions on how to review your proof when it is ready. Please keep in mind that we are working through a large volume of accepted articles, so please give us a few days to review your paper and let you know the next and final steps.

Lastly, if your institution or institutions have a press office, please let them know about your upcoming paper now to help maximize its impact. If they'll be preparing press materials, please inform our press team within the next 48 hours. Your manuscript will remain under strict press embargo until 2 pm Eastern Time on the date of publication. For more information, please contact onepress@plos.org.

You will receive an invoice from PLOS for your publication fee after your manuscript has reached the completed accept phase. If you receive an email requesting payment before acceptance or for any other service, this may be a phishing scheme. Learn how to identify phishing emails and protect your accounts at https://explore.plos.org/phishing.

If we can help with anything else, please email us at customercare@plos.org.

Thank you for submitting your work to PLOS ONE and supporting open access.

Kind regards,

PLOS ONE Editorial Office Staff

on behalf of

Dr. Fumihiro Yamaguchi

Academic Editor

PLOS One
